# Multiple Pigment Cell Types Contribute to the Black, Blue, and Orange Ornaments of Male Guppies (*Poecilia reticulata*)

**DOI:** 10.1371/journal.pone.0085647

**Published:** 2014-01-22

**Authors:** Verena A. Kottler, Iris Koch, Matthias Flötenmeyer, Hisashi Hashimoto, Detlef Weigel, Christine Dreyer

**Affiliations:** 1 Department of Molecular Biology, Max Planck Institute for Developmental Biology, Tübingen, Germany; 2 Max Planck Institute for Developmental Biology, Tübingen, Germany; 3 Bioscience and Biotechnology Center, Nagoya University, Nagoya, Japan; Arizona State University, United States of America

## Abstract

The fitness of male guppies (*Poecilia reticulata*) highly depends on the size and number of their black, blue, and orange ornaments. Recently, progress has been made regarding the genetic mechanisms underlying male guppy pigment pattern formation, but we still know little about the pigment cell organization within these ornaments. Here, we investigate the pigment cell distribution within the black, blue, and orange trunk spots and selected fin color patterns of guppy males from three genetically divergent strains using transmission electron microscopy. We identified three types of pigment cells and found that at least two of these contribute to each color trait. Further, two pigment cell layers, one in the dermis and the other in the hypodermis, contribute to each trunk spot. The pigment cell organization within the black and orange trunk spots was similar between strains. The presence of iridophores in each of the investigated color traits is consistent with a key role for this pigment cell type in guppy color pattern formation.

## Introduction

The spectacular orange, yellow, white, and black along with the blue to green iridescent colors of male guppies (*Poecilia reticulata*) have attracted the attention of biologists and hobby breeders for almost a century [1]–[5]. The guppy is a small live-bearing freshwater fish native to northeastern South America. Guppy populations have been studied most extensively on the island of Trinidad, where male coloration, as well as other traits, such as body shape and life history characteristics, covary with predation intensity [6], [7].

Mate choice experiments have demonstrated that guppy females, which are camouflaged by an inconspicuous reticulate pattern [4], [8], prefer males with high amounts of orange and iridescent pigments [6], [9], [10]. Both orange and iridescent ornaments can indicate a male’s current physical condition and genetic quality. The orange spots contain two types of pigments, carotenoids, which are obtained from the food, mainly from unicellular algae, and pteridines, which are synthesized de novo [11], [12]. Orange pigments therefore reflect a male’s foraging efficiency and ability to synthesize pteridines [11]–[13]. Pteridine production within the orange spots of wild guppy males varies with carotenoid availability; for instance, males produce less pteridines in habitats in which carotenoids are scarce, leading to a relatively constant pteridines to carotenoids ratio, and hence orange hue, across populations [11], [12]. A recent study has shown that female guppy preference for this specific orange hue causes this pattern [14]. Iridescent ornaments increase the risk of being noticed by predators and hence provide information on a male’s capability to evade these [6], [9], [15]. Males also intensify their black pigmentation during courtship, which might emphasize orange areas [6], [16]. The amount and size of male ornaments is highly heritable and a substantial portion is inherited in a Y-linked manner from the father [2], [5], [8], [17], [18]. Studies have demonstrated that guppy females favor males with rare or novel color patterns over males with familiar phenotypes, suggesting that negative frequency-dependent selection contributes to the maintenance of male color polymorphisms within guppy populations [19]–[23].

**Figure 1:**
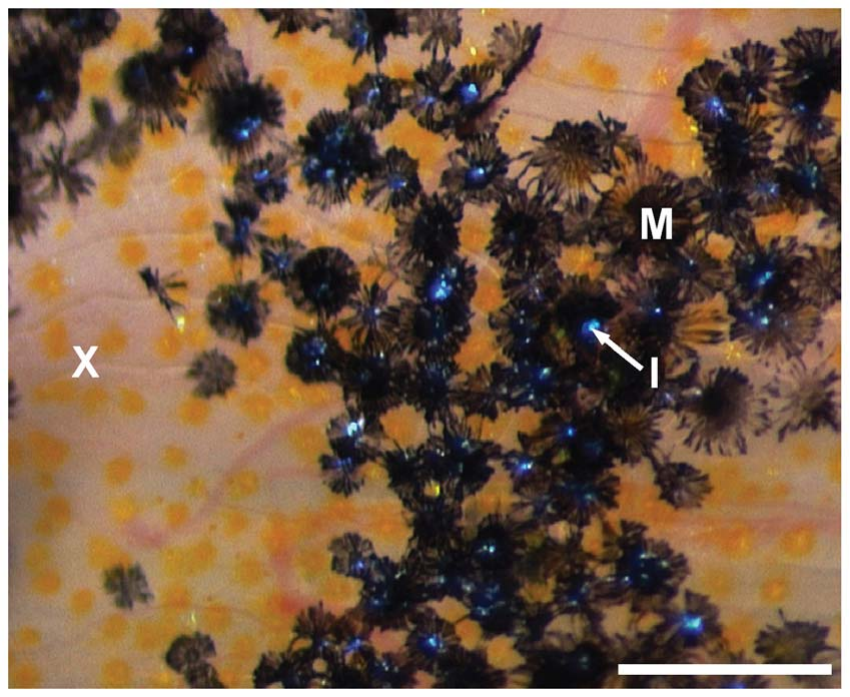
Pigment cell types of the guppy. Xanthophores (X), melanophores (M) and iridophores (I) on the dorsal side of a guppy female shown under incident light. Leucophores could not be identified. Scale bar: 200 µm.

While the selection pressures driving male color patterns have been well studied, little is known about the morphology of male ornaments. Five pigment cell types have been described in the skin of the guppy: black melanophores, orange to yellow xanthophores, red erythrophores, blue to green iridescent iridophores (Figure 1), and white leucophores [4], [8], [24]–[28]. The pigment organelles of melanophores, xanthophores, and erythrophores contain light-absorbing pigment colors, namely eumelanin and carotenoids/pteridines, respectively [11], [29]. The thin guanine crystals found in organelles within iridophores produce glittering blue, green, and silvery structural colors by thin film interference and refraction of incident light waves [26], [28], [30]. Leucophores appear whitish by scattering light in various directions; their pigment granules might contain uric acid [28], [30]–[32].

The precursors of vertebrate chromatophores migrate from the neural crest to various regions within the body [33], [34]. There is increasing evidence that both short- and long-range interactions between different types of pigment cells are required for proper migration, differentiation, and survival of their precursors. For example, during zebrafish (*Danio rerio*) pigment pattern development, iridophores stimulate xanthophore precursors to migrate to the prospective interstripe regions, but inhibit melanoblast localization to these areas, which then accumulate in the adjacent stripes [35]. Similarly, zebrafish xanthophores promote stripe development by short-range inhibitory and long-range stimulating interactions with melanophores [36]–[40]. Xanthophore-melanophore interactions are also crucial for the development of the male-specific pattern of the guppy, as male guppies lacking xanthophores due to a mutation in *colony-stimulating factor 1 receptor a* (*csf1ra*) also have severe melanophore localization defects [8]. To unravel such interactions in the guppy, it is critical to understand how different pigment cell types are organized within the male ornaments, which can be assessed best by transmission electron microscopy (TEM) [41]–[44]. Previous TEM studies on guppy pigment cells focused on the identification of different chromatophore types in the tail fins of adult males and on the development of lateral iridophores on the trunk [24]–[28]. The guppies investigated in these prior studies were obtained from pet shops. Unfortunately, the precise position of the ornaments on the guppy body was not documented, making it difficult to relate these TEM images to the macroscopically visible ornaments.

Here, we describe the pigment cell distribution, for which we subsequently use the term ultrastructure, within the blue, black, and orange trunk spots and fin color patterns of male wild-type guppies from three genetically divergent strains. TEM revealed that several chromatophore types contribute to each ornament. We could, however, not identify any leucophore. Our comprehensive study on pigment cell distribution in the skin of male guppies provides a foundation from which the natural variation in the placement and expression of male ornaments can be studied.

## Results and Discussion

### Identification of chromatophore types

**Figure 2:**
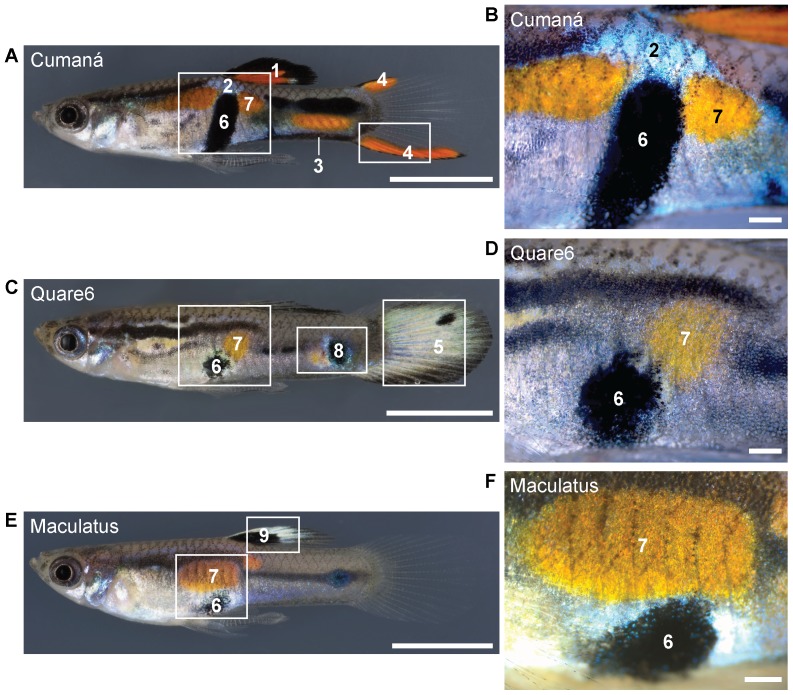
Phenotypes of male Cumaná, Quare6, and Maculatus guppies. (A,C,E) Lateral aspects of adult males taken under incident light conditions. White rectangles indicate details enlarged in (B,D,F) and Figures 6 and 7. Traits are labeled with numbers according to their appearance in the text: 1, Cumaná black and orange ornaments on the dorsal fin; 2, Cumaná blue iridescent spot; 3, Cumaná ventral black margin of the caudal peduncle; 4, Cumaná orange-black lining of the tail fin; 5, Quare6 tail fin color pattern; 6, central black spot; 7, central orange spot; 8, Quare6 posterior black spot on caudal peduncle; 9, Maculatus black spot and whitish ornaments on the dorsal fin. We investigated the ultrastructure of traits 2, 4 (orange part), 5, 6, 7, 8, and 9 (whitish part). Scale bars: (A,C,E) 0.5 cm; (B,D,F) 500 µm.

We investigated the pigment spots and fin color patterns of male Cumaná, Quare6, and Maculatus guppies. Cumaná guppies are derived from a wild population in Venezuela [45]; the inheritance of the black and orange ornaments on the dorsal fin, the blue iridescent spot on the trunk, and the ventral black margin of the caudal peduncle is linked to the male Y chromosome (Figure 2A, 2B) [17], [18], [46]. Quare6 guppies are descendants of fish from the Quare river on Trinidad [47]. Male Quare6 guppies display roundish black and orange spots on their body and older males often develop brilliant color patterns on the tail fin (Figure 2C, 2D) [8], [17]. Maculatus guppies have been bred in captivity by researchers and hobby breeders for almost a hundred years [1], [48]; their central black and orange spots on the trunk in combination with the black spot on the dorsal fin are Y-linked (Figure 2E, 2F) [1]. The black in the dorsal fin of Maculatus males is usually surrounded by whitish pigments (Figure 2E) (VAK and CD, unpublished data and [48]).

We analyzed the ultrastructure of the central orange and central black spot near the gonopodium, as these two spots are present in all three strains despite their considerably different male ornaments (Figure 2) [8]. Additionally, we included the Cumaná blue iridescent spot and some typical color fin patterns of the three strains into our analysis (Figure 2). Our aim was to identify the different types of pigment cells, to clarify how they are organized within the skin, and to determine whether the ultrastructure of the central orange and black spots are similar in these strains.

**Figure 3:**
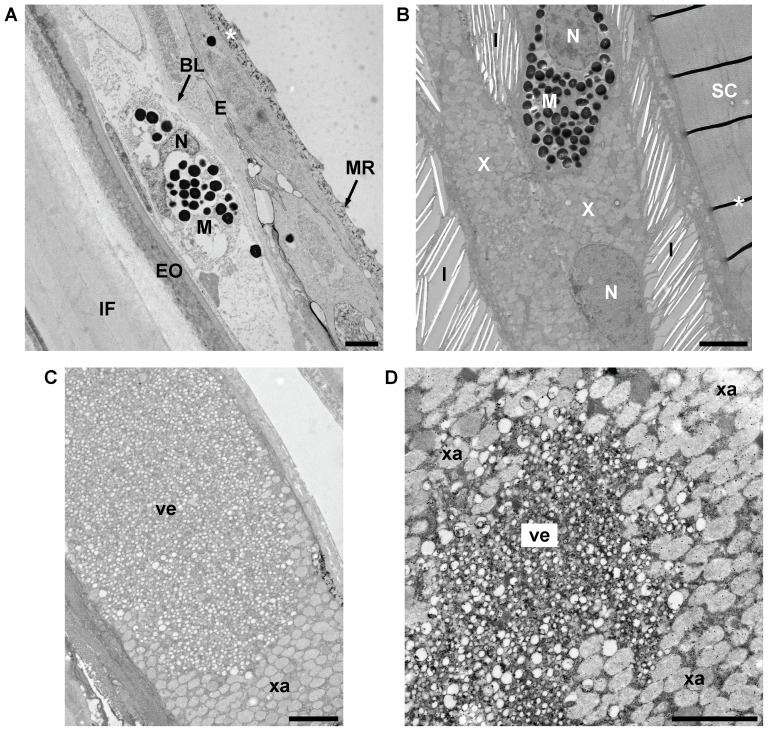
TEM images of guppy chromatophore types. (A) Melanophore on top of a scale in the dermis. Melanophores can be recognized by their dark-appearing pigment organelles, the eumelanin-containing melanosomes. (B) Melanophores, xanthophores, and iridophores in the hypodermis of the central orange spot of a Maculatus male. (C,D) Dermal xanthophores within the central orange spot of a Cumaná male. BL, basal lamina demarcating the boundary between the epidermis and dermis; E, epidermis; EO, external osseous layer of scale; I, iridophore; IF, internal fibrillary plate of scale; M, melanophore; MR, microridges of the epidermis; N, nucleus; SC, stratum compactum of dermis; ve, small vesicles or granules described in the text; X, xanthophore; xa, xanthosomes. Asterisks exemplarily mark artifacts caused by sample preparation; inflated empty spaces within iridophores are not marked. Individuals from which images (B-D) were taken were post-fixed with osmium tetroxide. Scale bars: 2 µm.

**Figure 4:**
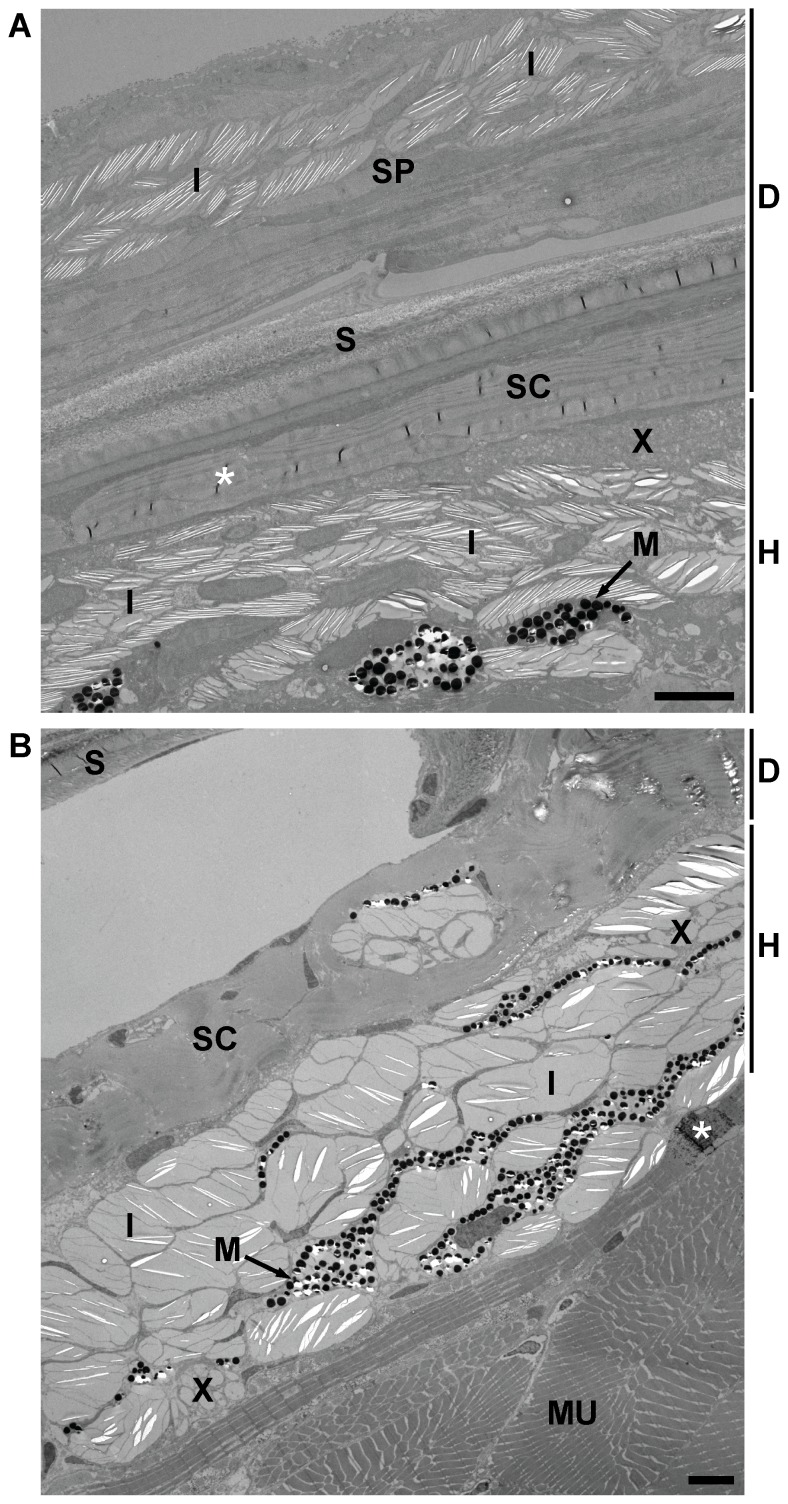
Ultrastructure of Cumaná blue spot. (A,B) TEM images of Cumaná blue spot. An image of the blue spot taken under incident light conditions is shown in Figure 2B (trait 2). Dermal iridophores and hypodermal iridophores and melanophores contribute to the spot. The epidermis was detached during sample preparation in (A). D, dermis; H, hypodermis; MU, muscle; S, scale; SP, stratum spongiosum of dermis. For other abbreviations see Figure 3. Individual from which image (A) was taken was post-fixed with osmium tetroxide. Scale bars: 5 µm.

Previously, two types of melanophores were described in wild-type guppies: dendritic ones, which are located on top of the scales, and corolla ones, which are located more deeply in the skin [4], [8]. When guppy scales are removed, dendritic melanophores associated with them are usually detached as well (VAK, unpublished data and [5]). Consistently, we detected melanophores in the epidermis and dermis covering the scales as well as in the dermis and hypodermis beneath the scales by TEM (Figures 3A, 4). The differentiation or survival of the superficial dendritic melanophores of the guppy depends on the type III receptor tyrosine kinase Kita, as there are less dendritic melanophores in the guppy *kita* mutant golden [3]–[5], [8]. The corolla melanophores in the deeper dermis and hypodermis were frequently associated with iridophores (Figure 1; shown in more detail in Figure 4). A subpopulation of these melanophores of the guppy, which appears early in development, depends on Kita as well [8]. As all males were euthanized with tricaine before fixation, the melanosomes within the melanophores should be mostly dispersed in our samples [49].

**Figure 5:**
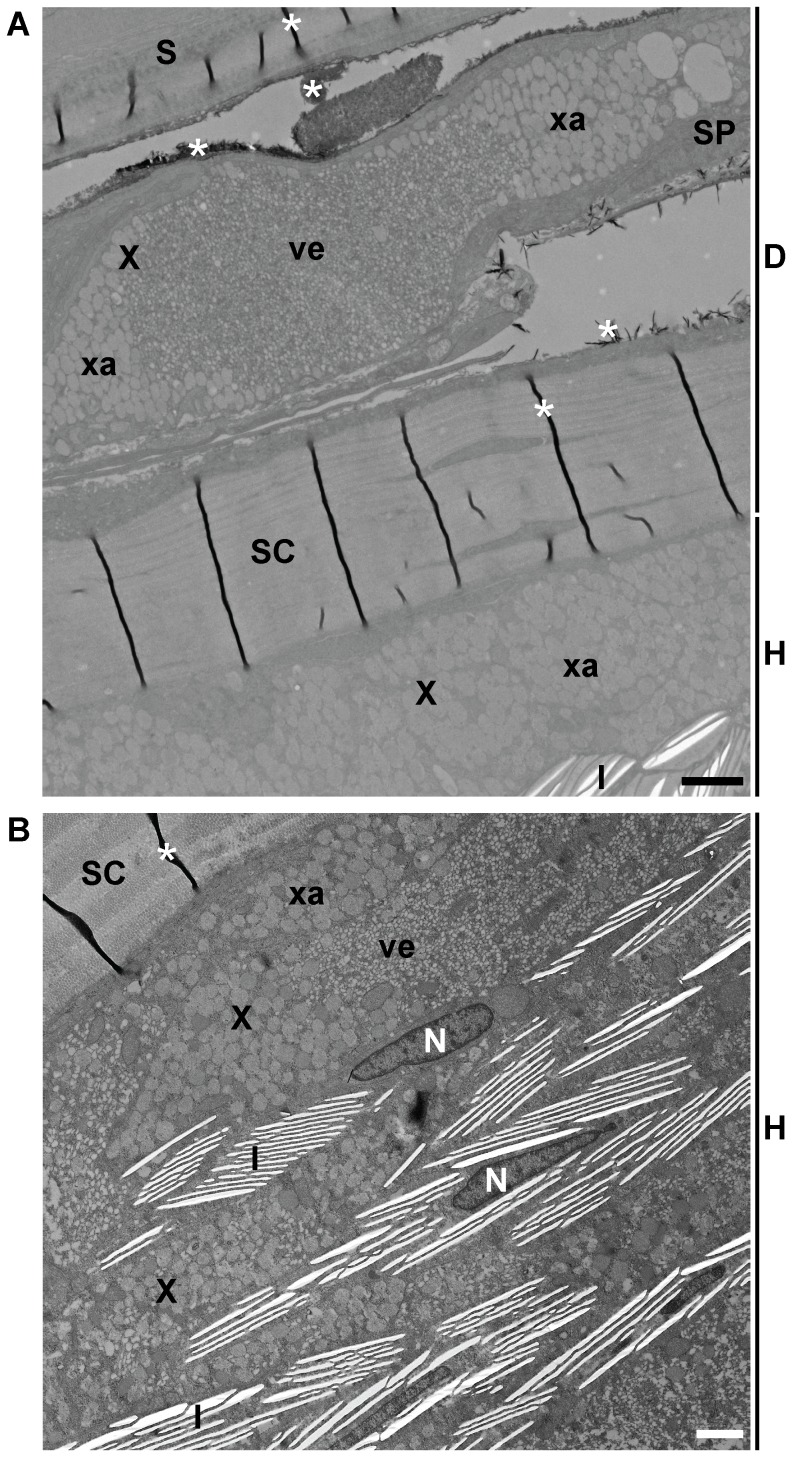
Ultrastructure of Cumaná central orange spot. (A,B) TEM images of Cumaná central orange spot. An overview image of the pigment cell distribution is shown in Figure S1. An image of the central orange spot taken under incident light conditions is shown in Figure 2B (trait 7). Dermal xanthophores and hypodermal xanthophores and iridophores contribute to the spot. For abbreviations see Figures 3 and 4. Individuals from which images were taken were post-fixed with osmium tetroxide. Scale bars: (A) 2 µm; (B) 1 µm.

**Figure 6:**
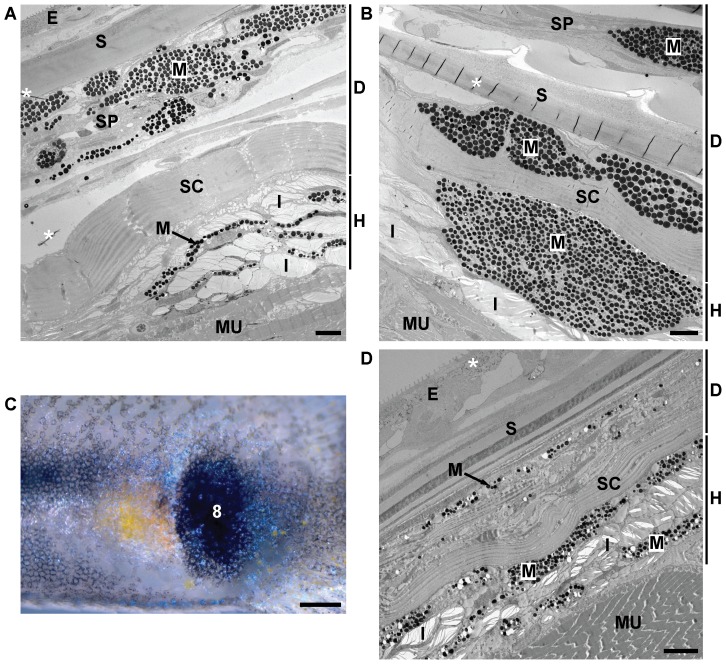
Ultrastructure of Cumaná central black spot and Quare6 posterior black spot. (A,B) TEM images of Cumaná central black spot. An image of the central black spot taken under incident light conditions is shown in Figure 2B (trait 6). Melanophores in the dermis and melanophores and iridophores in the hypodermis contribute to the spot. (C) Detail image of area boxed in Figure 2C showing Quare6 ornaments on the lateral caudal peduncle. Image was taken under incident light conditions. 8, posterior black spot. (D) TEM image of Quare6 posterior black spot. Individual from which image (B) was taken was post-fixed with osmium tetroxide. For abbreviations see Figures 3 and 4. Scale bars: (A,B,D) 5 µm; (C) 500 µm.

**Figure 7:**
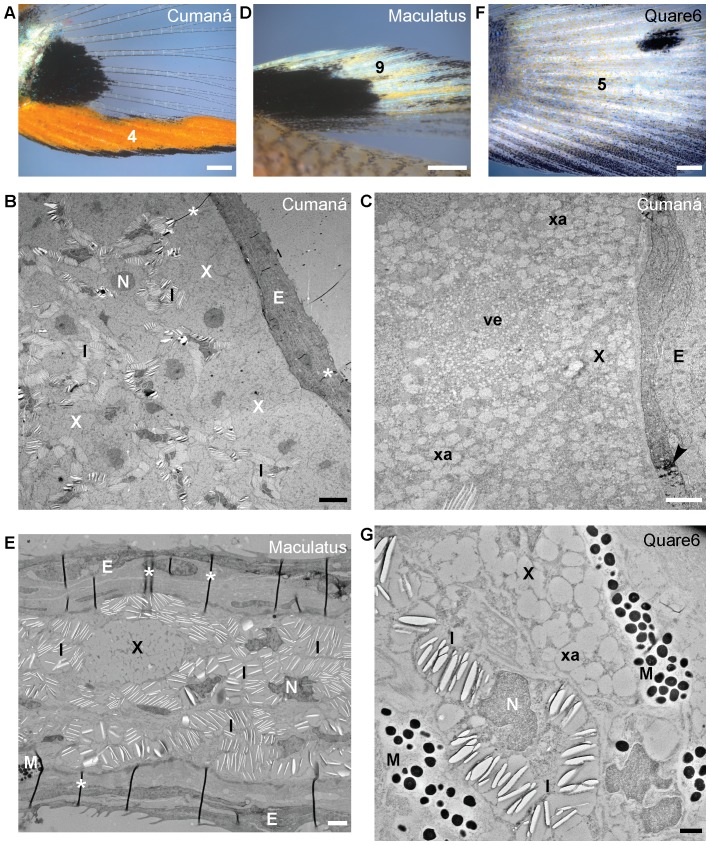
Ultrastructure of fin color patterns. (A,D,F) Detail images of regions boxed in Figure 2A, 2C, and 2E taken under incident light conditions. (D) and (F) are from the individual shown in Figure 2E and 2C, respectively. (A) is from a Cumaná male different from the one shown in Figure 2A. (B,C,E,G) TEM images. (A) Cumaná orange-black margin of tail fin (trait 4). The ultrastructure of the orange part is shown in (B) and (C). (D) Maculatus dorsal fin ornaments (trait 9). The ultrastructure of the whitish part is shown in (E). (F) Quare6 tail fin pattern (trait 5). The ultrastructure of the whitish area is shown in (G). For abbreviations see Figures 3 and 4. Individual from which image (C) was taken was post-fixed with osmium tetroxide. Scale bars: (A,D,F) 500 µm; (B) 5 µm; (C,G) 1 µm; (E) 2 µm.

We found iridophores in both dermal and hypodermal skin layers (Figures 3B, 4, 5, 6A, 6B, 6D, S1, S2A-C, S3). They contain stacked guanine crystals, called ‘reflecting platelets’ [28], [31]. The crystals usually are lost during sample preparation for TEM, leaving empty spaces that appear inflated in the TEM images (Figures 3B, 4, 5, 6A, 6B, 6D, 7B, 7E, 7G, S1, S2A-C, S3) [26]. The color produced by the iridophores highly depends on the orientation of the platelets relative to each other and the epidermis, as discussed in more detail below [26], [30], [31]. The number and distance between platelets, and the thickness of the cytoplasm influences their reflection as well [26], [30], [31]. We did not try to measure the size of the reflecting platelets or the thickness of the cytoplasm between them, as we hardly found any intact ones and the samples were affected by cytoplasmic shrinkage due to sample preparation [50]. The size of the platelets also varied greatly depending on their orientation with respect to the plane of section (Figures 3B, 4, 5, 6A, 6B, 6D, 7B, 7E, 7G, S1, S2A-C, S3).

Xanthophores were identified by the xanthosomes, which are roundish pigment organelles that appear clear or of medium electron density and are approximately 0.5 µm in diameter (Figures 3C, 3D, 4, 5, 7B-G, S1, S2) [27], [32], [42], [43], [51], [52]. Xanthophores were found in both the dermis and hypodermis (Figures 3C, 3D, 4, 5, S1, S2). The proliferation and/or dispersion of these cells during guppy development depends on signaling through the type III receptor tyrosine kinase Colony-stimulating factor 1 receptor a (Csf1ra) [8]. Previously, yellow to orange xanthophores and red erythrophores were described in guppy skin [4], [8], [27]. The discrimination between xanthophores and erythrophores, however, is solely based on the apparent color, not on structural differences [30]; therefore some authors have called them xantho-erythrophores [9], [53]. We use the term xanthophore here.

Some xanthophores contained clusters of small, light-appearing vesicles or granules in addition to the considerably larger xanthosomes (Figures 3C, 3D, 5, 7C, S2A, S2B, S2D). We found such clusters in some xanthophores within the central orange spot of males from all three strains and in some xanthophores within the orange part of the orange-black margin of the Cumaná tail fin (Figures 3C, 3D, 5, 7C, S2A, S2B, S2D). It has been previously speculated that these clusters might be involved in carotenoid accumulation within the xanthophores [27]. Carotenoid droplets with an approximate diameter of 0.1 to 0.3 µm have been described in medaka (*Oryzias latipes*) and the teleost *Trematomus bernacchii* [52], [54]. The organelles that we observed had an approximate diameter of 0.14 µm (Figures 3C, 3D, 5, 7C, S2A, S2B, S2D). Whether all guppy xanthophores contain such small additional organelles or whether these clusters are associated with a special developmental stage or location of the xanthophores is unknown.

The leucophores that have been previously described in the guppy contained globular leucosomes with a diameter of approximately 0.5 to 0.8 µm. Unfortunately, the study describing these leucosomes did not include any xanthophore images for comparison [28]. The leucosomes of medaka and the killifish *Fundulus heteroclitus* are supposed to be of approximately the same size as the ones of the guppy [32], [55]. We did not observe any other cells resembling chromatophores beside the described melanophores, iridophores, and xanthophores. Interestingly, some larval chromatophores of medaka contain reddish pigment that they lose during further development, thereby becoming whitish leucophores [32], [56]. This raises the possibility that some organelles can contain several pigment types and might change in the course of development [56], [57]. Moreover, pigment cells containing two types of pigment organelles, so-called ‘dichromatic chromatophores’, have been described in some vertebrates. Examples of such chromatophores are the cyano-erythrophores of the mandarin fish (*Synchiropus splendidus*) and the erythro-iridophores of the diadema pseudochromis (*Pseudochromis diadema*) [58], [59]. The organelles that we observed within the guppy xanthophores resemble leucosomes except for their smaller size. Further studies will elucidate whether they are carotenoid granules or leucophore-specific organelles containing uric acid.

### Ultrastructure of Cumaná blue spot

While the iridescent areas of Quare6 and Maculatus males are largely diffuse, Cumaná males always have a distinct bluish iridescent spot below the dorsal fin (Figure 2A, 2B) [17], [18], [46]. Under certain light conditions, especially when seen from above, this spot might also appear whitish (data not shown). Inspection of TEM images revealed that this spot is formed by two sheets of iridophores, one of which is located in the stratum spongiosum of the dermis and the other in the hypodermis (Figure 4). Just below the hypodermal iridophores, on top of the muscles, we found melanophores whose appendices frequently protruded into the iridophore layer (Figure 4). The melanophores did not form a complete sheet; in some areas the iridophores were in direct contact with the underlying muscle layer (Figure 4). Melanophores were also present within the dermal iridophore layer (data not shown). The hypodermal as well as the dermal iridophore sheet contained some xanthophores, too (Figure 4 and data not shown). The reflecting platelets of the iridophores appeared to be randomly distributed and were tilted in different directions at some locations, whereas they looked more organized at other locations (Figure 4).

A previous study on the development of iridophores on the lateral trunk of fancy guppies reported that all reflecting platelets had an angle of approximately 15–30° relative to the surface of the fish, thought to account for the blue-green reflection with a wavelength of 496 nm [26]. The light blue coloration of the common surgeonfish (*Paracanthurus hepatus*) is derived from a double layer of iridophores in which the reflective platelets are oriented almost in parallel relative to the fish surface; the iridophores are located on top of melanophores [60]. Ordered iridophores above melanophores have also been observed in the blue skin of the blue-green damselfish (*Chromis viridis*) and the lizard *Plestiodon latiscutatus* [44], [61]. In contrast, randomly arranged reflecting platelets usually seem to produce a whitish coloration, e.g. in the white spots of the domino damsel (*Dascyllus trimaculatus*) [41]. We found that both disordered and ordered reflecting platelets are present within the bluish to whitish spot of Cumaná males. The appearance of this spot is dynamic and depends on the angle of the incident light and the movement of the fish. The bluish coloration is presumably derived from the platelets that are arranged in parallel, while the whitish color comes from the disordered platelets. Interestingly, melanophores contribute to the ultrastructure of the blue ornament of the Cumaná guppy like in the common surgeonfish, the blue-green damselfish, and *P. latiscutatus*. Even within the stratum compactum, a melanophore was found in close contact with an iridophore (Figure 4B). We suspect that the melanophores modulate the reflection of the iridophores.

### Ultrastructure of central orange spot

We detected large accumulations of xanthophores in the stratum spongiosum of the dermis and hypodermis within the Cumaná central orange spot (Figures 5, S1). These xanthophores frequently contained clusters of the small vesicles or granules described above (Figure 5). Between the xanthophores in the hypodermis were numerous iridophores, with reflecting platelets aligned in parallel (Figures 5B, S1). They were arranged slightly obliquely relative to the epidermis (Figure S1). Additionally, some melanophores, as well as iridophores with a more random arrangement of platelets, were present in the hypodermis (data not shown). We also found some iridophores and melanophores in the dermis (Figure S1 and data not shown). The ultrastructure of the central orange spot of Quare6 and Maculatus males appeared similar, except that we found none or only very few xanthophores and iridophores in the dermis (Figure S2A, S2C).

In general, the orange areas of Cumaná males appear darker and more intense than the orange areas of Quare6 and Maculatus males (Figure 2 and data not shown). As all of our fish are fed with *Artemia*, hence uptake *Artemia* carotenoids, the reasons for this must be intrinsic. Our results indicate that thick xanthophore layers in both the dermis and hypodermis cause the intense orange coloration of Cumaná males. The central orange spots of Quare6 and Maculatus males seem to contain fewer xanthophores as the more superficial xanthophore layer is mostly absent. This might be the reason why the spots of Quare6 and Maculatus males appear yellower than the ones of Cumaná males. Alternative explanations are that the production of pteridines varies between the strains as shown for other guppy populations [14], or that the uptake and metabolism of the *Artemia* carotenoids differs, leading to a different pteridines to carotenoids ratio. So far, only the pteridines synthesized by male guppies from Trinidad, which are drosopterins, have been positively identified [11]. As we analyzed the ultrastructure of the central orange spot of only four Cumaná males, more individuals need to be investigated to clarify whether indeed all Cumaná males have an additional superficial xanthophore layer.

Large reflecting platelets that are ordered in parallel to the epidermis and are not underlain with melanophores usually produce silvery, mirror-like reflections [30], [42], [62]. The numerous iridophores in the hypodermis of the male central orange spot of the guppy form reflective sheets. These might reflect light that has not been absorbed or reflected before by the xanthophores, which would make the male orange ornaments shinier and probably more attractive for females by combining orange with iridescence coloration [6], [9]. The observed differences in iridophores might also contribute to the color differences of the orange areas.

The ultrastructure of the central orange spot superficially resembles the one observed in the yellowish interstripes of zebrafish, yet the zebrafish xanthophores and iridophores are distributed exclusively in the hypodermis [42]. Whether xanthophore-iridophore interactions like the ones in zebrafish are required for the formation of the central orange spot of the guppy still needs to be investigated [35].

### Ultrastructure of central black spot

We found two layers of melanophores within the central black spot of Cumaná, Quare6, and Maculatus males, which were located in the stratum spongiosum of the dermis and in the hypodermis (Figures 6A, 6B, S3). The melanophores were very large, occasionally up to 100 µm in diameter. The ones in the hypodermis were intermingled with iridophores, whose reflecting platelets seemed to be randomly arranged (Figures 6A, 6B, S3). Some xanthophores were scattered in the melanophore layers as well (data not shown).

Since iridophores cause the whitish belly coloration of fish including the guppy ([41] and data not shown), we considered the possibility that iridophores are merely present within the central black spot because this ornament is located on the lateral part of the belly. To test this, we investigated the ultrastructure of the posterior black spot located on the caudal peduncle of Quare6 males (Figures 2C, 6C, 6D). Like in the central black spot, dermal melanophores and hypodermal melanophores and iridophores were present (Figure 6D), suggesting that iridophores are components of the black spots of guppy males independent of their location. Iridophores also contribute to the black and white eye spots seen on the caudal peduncle of some guppy strains, e.g. the BDZW1 strain, by forming a light circle around the black spot [8].

### Ultrastructure of fin color patterns

Patterns on the dorsal and tail fins vary greatly between the three guppy strains considered here (Figure 2). We found xanthophores and iridophores in the orange part of the orange-black margin of the Cumaná tail fin and the whitish-yellow part of the dorsal fin of Maculatus males (Figure 7A-E). The reflecting platelets appeared to be randomly oriented (Figure 7B, 7E). While xanthophores were more abundant in the orange Cumaná tail fin margin, iridophores were more frequent in the white ornament on the Maculatus dorsal fin (Figure 7B, 7C, 7E). The xanthophores within the orange Cumaná tail fin margin frequently contained the small vesicles or granules beside xanthosomes (Figure 7C). The white part of the Maculatus dorsal fin also contained few melanophores (Figure 7D and data not shown). Xanthophores and iridophores are also associated with each other in the light stripe regions of zebrafish fins [43]. Iridophores in the orange tail fin margin of Cumaná males might enhance the orange signal by increasing reflection. We found scattered iridophores, xanthophores, and melanophores in the tail fin of Quare6 males (Figure 7F, 7G). All fin pigment cells appeared to be located in hypodermal tissue similar to the situation in zebrafish fins [43].

## Conclusions and Outlook

Our study demonstrates that at least two of the three types of pigment cells contribute to each of the investigated ornamental traits of Cumaná, Quare6, and Maculatus guppy males, suggesting that complex interactions between different chromatophore types both may be involved in establishing color patterns and enhance color signals in these strains. Notably, the ultrastructure of the central orange and black spots of Cumaná, Quare6, and Maculatus males is very similar, despite Cumaná and Quare6 guppies being derived from geographically distant populations that may have been separated for almost a million years [63]. More individuals from other populations need to be investigated to confirm that the ultrastructure of these spots is indeed conserved within the guppy. Additionally, TEM in combination with spectrophotometry may help clarify the relationship between ultrastructure and spectral characteristics like hue, saturation, and lightness of guppy ornaments in the future. It would be especially interesting to investigate whether natural guppy populations that differ in the production of drosopterins and hence orange coloration [14] also show differences in the ultrastructure of their orange ornaments.

We have also shown that the pigment cells within the trunk ornaments form thick sheets in the dermis and hypodermis. This contrasts with the situation in zebrafish trunk stripes, in which the chromatophores are restricted to the hypodermis [42], [43]. While we could easily identify melanophores, xanthophores, and iridophores in all males, we were not able to confirm the presence of leucophores. Future studies testing the dispersion-aggregation response of guppy chromatophores might reveal whether pigment cells showing a reaction opposite to that of melanophores and xanthophores exist in the three investigated guppy strains. Such a response would be typical for leucophores [64].

Iridophores were a major component of all ornaments, even of ornaments perceived as orange or black. The close association of iridophores with melanophores, and of iridophores with xanthophores, raises the question whether iridophores might interact with xanthophores and melanophores during color pattern formation in male guppies, as has been shown for zebrafish [35]. Because iridophores are transparent and reflective, their photographic documentation highly depends on reproducible light conditions. This might be the reason why previous studies on guppy pigment mutants focused only on melanophore and xanthophore defects. Our study suggests that it is crucial to consider iridophores as well, which might attract melanophores and xanthophores to the locations where spots arise during male color pattern formation. Depending on the location, iridophores might also repulse xanthophores or melanophores, or influence their survival [35]. It would be most interesting to investigate whether iridophores accumulate at the sites of prospective black and orange spots in juvenile males before melanophores and xanthophores appear. If this were the case, it would support a model in which subtle differences in iridophore migration and differential interactions of iridophore populations with other chromatophore types modulate male color pattern formation, ultimately leading to the extraordinary variation of male guppy ornaments.

## Materials and Methods

### Ethics Statement

This study was carried out in strict accordance with the German Protection of Animals Act (§ 11 Abs. 1 Nr. 1 a und b TierSchG); all experiments were permitted by the Regierungspräsidium Tübingen (approval ID 35/9185.46). Fish were euthanized using 0.1% (w/v) tricaine (ethyl 3-aminobenzoate methanesulfonate salt) solution pH 7.

### Guppy strains and rearing conditions

All guppies were reared at 25°C in a 12-hour light and dark cycle and fed six days a week with *Artemia.* The Cumaná and Quare6 strains originated from individuals that were collected in Venezuela near Cumaná and in the Quare river on Trinidad in 2003 [45], [47]. The laboratory strain Maculatus was first described comprehensively in 1922 [1]. The males that we used in our study were at least four months old.

### Imaging under incident light conditions

Images of males were taken with a Canon EOS 10D digital camera with a Canon Macro Lens EF 100 mm. A Leica MZFLIII dissecting microscope connected to a Zeiss AxioCam HRc color camera was used to visualize details of the male ornaments; images were processed with AxioVision Software Release 4.7.2. The brightness and contrast of some images was adjusted with Adobe Photoshop Software version 12.1.

### Transmission electron microscopy

The ultrastructure of each ornamental trait was investigated in four Cumaná, three Quare6, and three Maculatus males. Sample preparation for transmission electron microscopy was similar to [65]. Briefly, guppy males were sacrificed and fixed in 100 mM PO_4_ buffer pH 7.2 containing 4% formaldehyde and 2.5% glutaraldehyde at 4°C overnight. The fixated fish were dissected into small pieces according to the regions of interest. Subsequently, some of the samples were post-fixed with 1% osmium tetroxide on ice for one hour. All samples were then stained with 1% aqueous uranyl acetate at 4°C for one hour in the dark. The samples were dehydrated in a graded series of ethanol/water concentrations; subsequently, a graded series of epon/araldite resin (Araldite 502/Embed 812 Kit, EMS) in propylene oxide was used for embedding. Ultra thin sections of 70–100 nm of the samples were taken along the longitudinal axis of the fish using a Leica Ultracut UCT microtome. Specimens were examined in a FEI Tecnai G^2^ Spirit transmission electron microscope operating at 120 kV. Images were taken with a Gatan Ultrascan 4000 camera at maximum resolution using the manufacturer’s software. Adobe Photoshop Software version 12.1 was used to adjust the brightness and contrast of some images.

### Identification of skin layers

Skin layers were named according to [66].

### Measurements of organelle size

Diameters of the organelles found in clusters within xanthophores were measured with ImageJ 1.47 (rsbweb.nih.gov/ij/). We measured the diameter at the widest part of 100 organelles and calculated the average. These measurements are just an approximation, as the organelles were cross-sectioned at different planes.

## Supporting Information

Figure S1 **Overview TEM image of Cumaná central orange spot.** For abbreviations see Figures 3 and 4. Individual from which image was taken was post-fixed with osmium tetroxide. Scale bar: 10 µm.
(TIF)

Figure S2 **Ultrastructure of Quare6 and Maculatus central orange spots.** (A,B) TEM images of Quare6 central orange spot. (B) is an enlarged detail of (A) showing xanthosomes and vesicles or granules within a xanthophore as described in the text. (C,D) TEM images of Maculatus central orange spot. (D) shows xanthosomes and vesicles or granules within a xanthophore. The epidermis was lost during sample preparation. Images of the Quare6 and Maculatus central orange spots taken under incident light conditions are shown in Figure 2D and 2F (trait 7), respectively. For abbreviations see Figures 3 and 4. Individual from which images (A) and (B) were taken was post-fixed with osmium tetroxide. Scale bars: (A) 2 µm; (B,D) 1 µm; (C) 5 µm.
(TIF)

Figure S3 **Ultrastructure of Quare6 and Maculatus central black spots.** (A) TEM image of Quare6 central black spot. (B) TEM image of Maculatus central black spot. Images of the Quare6 and Maculatus central black spots taken under incident light conditions are shown in Figure 2D and 2F (trait 6), respectively. For abbreviations see Figures 3 and 4. Individuals from which images were taken were post-fixed with osmium tetroxide. Scale bars: 10 µm.
(TIF)
