# Supplementary figures and images for: Multiple Pigment Cell Types Contribute to the Black, Blue, and Orange Ornaments of Male Guppies (Poecilia reticulata)
Source: PLoS One. 2014 Jan 22;9(1):e85647. doi: 10.1371/journal.pone.0085647 (PMC3899072; doi:10.1371/journal.pone.0085647)

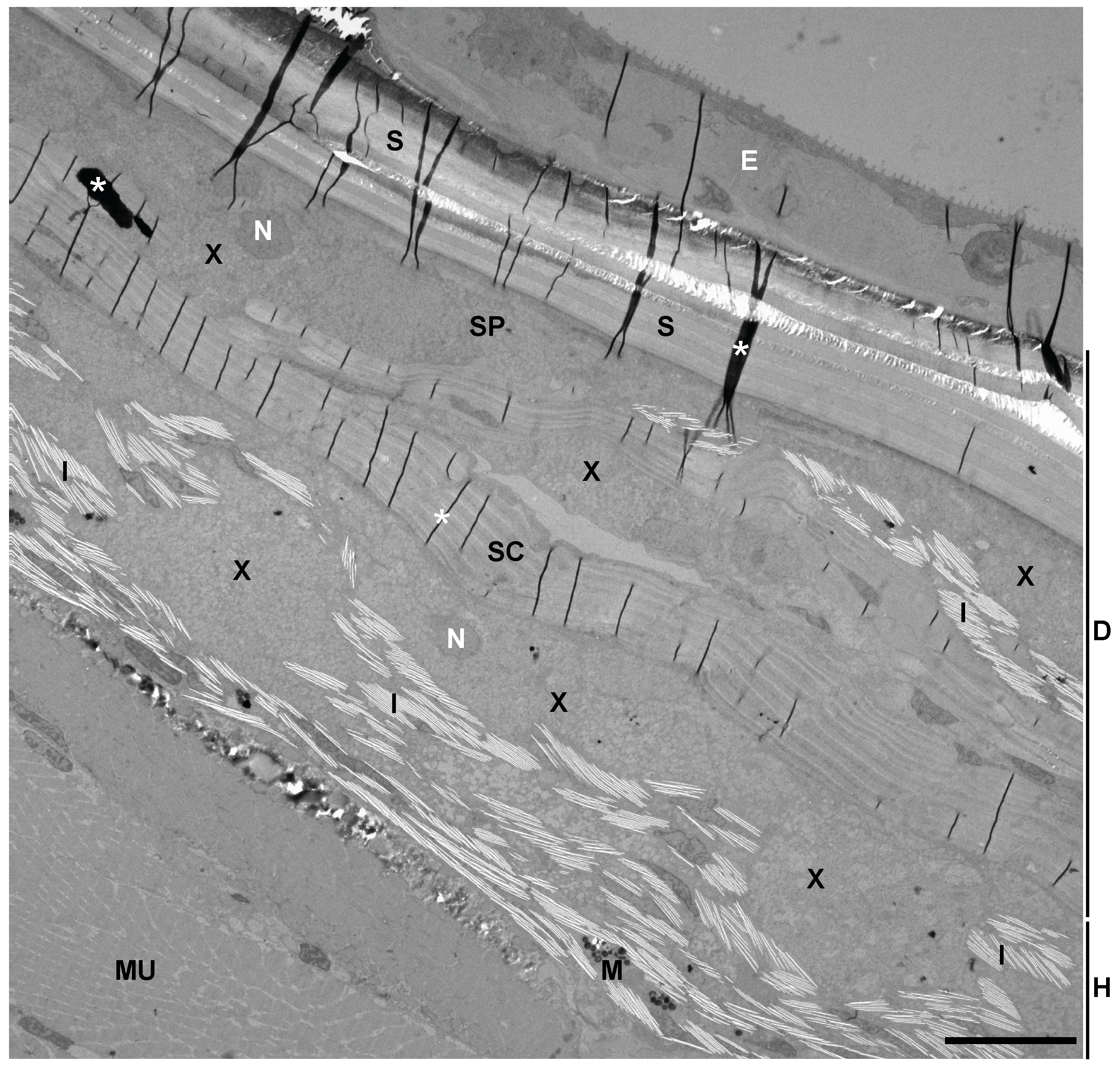

Supplement: Figure S1 — Overview TEM image of Cumaná central orange spot. For abbreviations see Figures 3 and 4. Individual from which image was taken was post-fixed with osmium tetroxide. Scale bar: 10 µm. (TIF) [file pone.0085647.s001.tif]

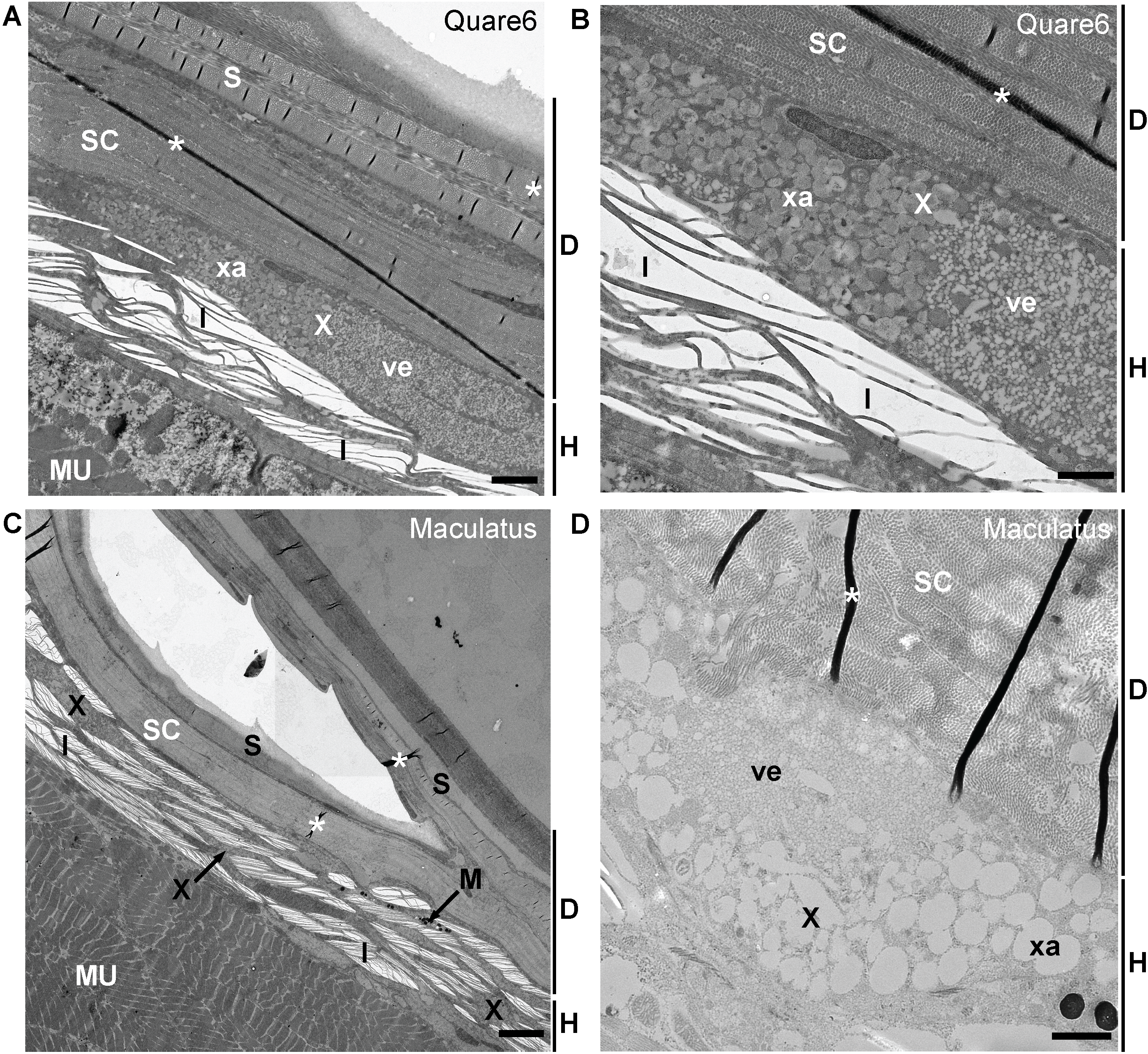

Supplement: Figure S2 — Ultrastructure of Quare6 and Maculatus central orange spots. (A,B) TEM images of Quare6 central orange spot. (B) is an enlarged detail of (A) showing xanthosomes and vesicles or granules within a xanthophore as described in the text. (C,D) TEM images of Maculatus central orange spot. (D) shows xanthosomes and vesicles or granules within a xanthophore. The epidermis was lost during sample preparation. Images of the Quare6 and Maculatus central orange spots taken under incident light conditions are shown in Figure 2D and 2F (trait 7), respectively. For abbreviations see Figures 3 and 4. Individual from which images (A) and (B) were taken was post-fixed with osmium tetroxide. Scale bars: (A) 2 µm; (B,D) 1 µm; (C) 5 µm. (TIF) [file pone.0085647.s002.tif]

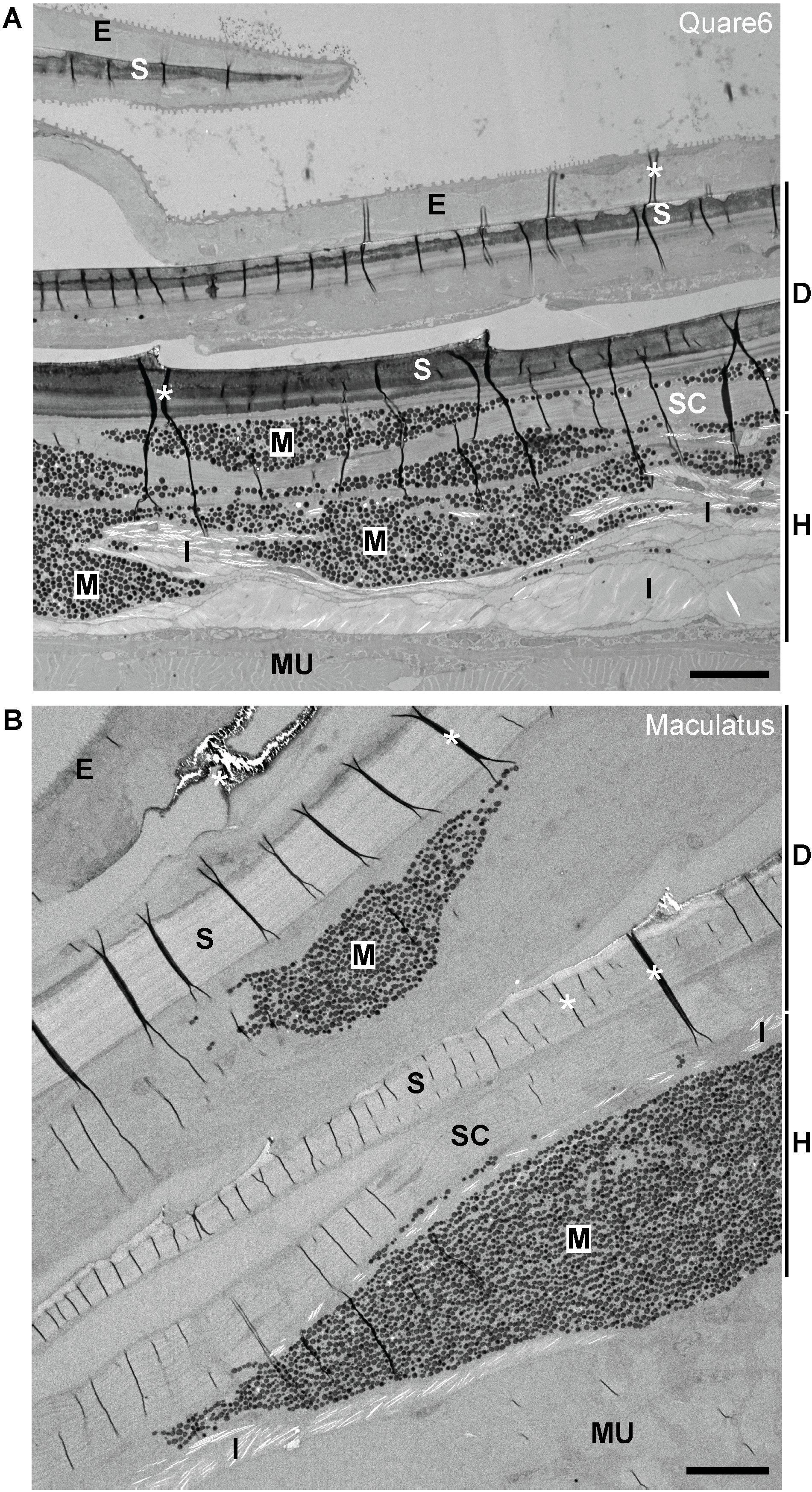

Supplement: Figure S3 — Ultrastructure of Quare6 and Maculatus central black spots. (A) TEM image of Quare6 central black spot. (B) TEM image of Maculatus central black spot. Images of the Quare6 and Maculatus central black spots taken under incident light conditions are shown in Figure 2D and 2F (trait 6), respectively. For abbreviations see Figures 3 and 4. Individuals from which images were taken were post-fixed with osmium tetroxide. Scale bars: 10 µm. (TIF) [file pone.0085647.s003.tif]
